# Observations on Nervous Disorders

**Published:** 1809-09

**Authors:** 


"Our readers will perceive that though the following is not the production of a Medical Writer, yet, that it is written by a man of science, who felt in himself so many of the symptoms as to induce him to watch it carefully in others. The descriptions are at once lively, impressive, and correct."

### Observations on Nervous Disorders. By the Rev. Mr. Patrick.

FEW works published by medical writers are so unsatisfactory and so defective in complete information, as those which have attempted to elucidate the subtle subject of nervous complaints. The anatomy of the brain, and the peculiar mode in which mind acts upon matter and body, escape the most sagacious Inquirer. Medical men, as they are rarely nervous, cannot speak the clear language of personal experience. Were I anxious to investigate the mechanism of a watch, I would listen to the experienced knowledge of a veteran watch-maker, not to the theories of a Cantabrigian lecturer on the mechanical powers.

On entering upon so delicate a subject, I tremble lest a fancy too animated, descriptions too lively, or language too faithfully picturesque, should increase the numbers of the nervous patients, or should hurry into that state of mental disease, those, to whom Nature has given a tendency and a mournful fitness for so wonderful an affection.

Let such readers either close this Essay at its first page, or should a rash curiosity lead them to peruse the whole, let their memory in future life dwell on the precautions and preventives, with which the Essay will conclude, and on the remedies which it will copiously and humanely prescribe; remedies on which a successful practice has stamped an indisputable value.

While the muscles and the fibres are the obvious, the nerves, which are of a similar nature, are the more delicate and secret portions of the body, which give to it all its locomotive powers, and impart to the brain its action of thinking. Their anatomical delineation may be read with every advantage in the Encyclopedia Britannica, under the article "nerves and nervous complaints." The nerves, like the Eolian harp, tremble at every vibration from external objects, and flutter at every collision of thought. They are the most delicate subdivision of our animal economy. They are too sensitive, and too irritable. I mean by irritation, neither furious passion, nor dark revenge; but a tendency to excessive excitement, and an agitation too extravagant for its apparent cause. Such patients are attacked attacked by a sensibility as keen as the sensibility of the infant, but it is unhappily accompanied with the reflecting powers of the man. They confess their weakness; they discover their want of judgment, or rather their want of firmness; yet the consciousness of such a frailty rather adds to their wretched sensation than rouses them to attempt their own cure. A respectable lady, who eventually died from a lethargy and a dropsy, superinduced by a tedious nervous affection, which had harrassed her during 30 years, assured the writer of this essay, that all who labour under a tendency to these curious disorders, may verify their own feelings, and may detect their own malady in the following description. "Some have hours of deep and awful grief; melancholy overspreads the soul; there is an appetite for retirement; for the house of the way-faring man in the wilderness; the soul is struggling through a mist; it feels its own large capacity for happiness, but happiness is denied to it." In different constitutions, and at different ages, some variety in the symptoms appear, but in all the patients an extraordinary, unexpected, and benumbing depression of spirits is experienced.

The grandfather of one of my early companions was so *loaded* and *overwhelmed* by this sensation that he blessed God, whenever the gout seized him, because it stimulated him with a very different feeling," and delivered him from its uniform and intolerable pressure. These two disorders alternately assailed him. No words, however poetical, and no images, however highly figurative, can paint to one who has not endured the torture, the peculiar weight and over-awing nature of this *mental chillness*, of this spiritual frost, which binds the cheerful current of thinking, and bends down the stubborn and reluctant mind to contemplate its present miseries alone, its immediate, its actual oppression. I have seen a lady, seated in her chair, motionless and apparently insensible; stupified with her own miseries; bending her eyes in terrible musing on the floor; the world lost to her; no friend, no sympathising relation, not even her aged father, able to draw from her a look, a word of consolation. In the midst of his own miseries, he tried, by the power of sacred music, to awaken her absorbed attention, and to recall her from her pains: a faint sigh alone conveyed to him her answer and her anguish. When such a wearisome weight of woe attacks the man of a firm mind and a robust frame, you perceive him anticipating the same *torpifying* sensation, as it spreads over his faculties and his soul, like a thick and sluggish cloud effacing the light of day. I saw a studious man, under these awful visitations, rise from his table, and as it were, feel after his understanding; struggle to retain his coolness of judgment, and his sober discernment, but struggle in vain; mind was lost to him for a few moments; he had forgotten his business, his place, his present circumstances; his soul was inveloped in one dark cloud; he neither reflected on the past, nor indulged in hopes of the future; he only experienced the galling and bitter misery of the minute; this sentiment, and this sensation, was his only feeling, his only contemplation.

I knew another who attempted to convince himself that he had not lost his reason, and fortunately attempted *to count his ten fingers*. This singular employment gave the learned author a melancholy assurance that all mind was not taken from him, and partially relieved him from the *solitary* thoughts of his present distress. Madness is not a disorder of a nervous nature, nor attended with nervous symptoms; for it is a total delirium, a day-dream, an absence of consciousness. But the peculiar and discriminating misery of the nervous patient consists in feeling his own miseries, in the ability to recollect them, and in the oppressing conviction of his inability to overcome at the moment the present and acrid torment in the mind. Superficial writers and unexperienced physicians err upon these remarkable points of difference between the cases. I have dwelt at greater length upon this symptom, as it continues in some patients through many tedious years. To delineate with precision the symptoms, the origin, and the progress of the disorder in individual patients, it is necessary to give a summary view of their biography, and of their bodily frame.

My intimate friend, Mrs. G———, the wife of a clergyman, who married her from attachment, and treated her from early youth to a very advanced age with the fondest affection, and the most studied attention, fell insensibly into the wretched agony of nervous depression. Her figure was broad, her limbs square and fully formed; her eye mild, her temper placid; her society engaging, and her original disposition cheerful, but not so in the extreme; neither hysterical in her feelings, nor wild in her imagination. An office for life-insurances would have required a very low premium for her chance; a philosopher would have calculated upon her continued tranquillity. Vain hopes, and delusive estimates; she remembered that her first tendency towards a nervous affection arose from the treatment reatment to which she was exposed under a father, passionate, boisterous, rude, and despotic. His sallies of fury approached to a frenzy, and gave her infant frame those tremblings and shudderings which, probably, relaxed all her system and unnerved her mind. The second cause arose from the solitary life which she spent in a forlorn village on the banks of the Humber, during the first seven years of her marriage. Habituated to the tumult or the activity of a seaport, she confessed that the poets described in colours very false in her estimate of happiness, the dull repose and heavy stillness of a village of peasants, who "slowly plodded their way," amidst a Dutch-like country, and its stupid hedges. The horrible uniformity of so wearisome a residence, gradually wore down her mind to gloomy musings, and to the discontented images of her sombrous life. At this critical moment, her only daughter, all her pride and hope, when she had completed her education in France and returned to London, on her journey home, died in the capital after an illness of three days; at the same moment her sun set in night. Nothing remained to her, she remarked to me, but a sure return of misery when she contemplated the desolate and childless situation. In vain her numerous friends offered their sympathy and confidence. Her soul refused comfort. The dead never return, and she saw that nothing remained to her but languishment and sorrow. From that bitter hour to the night of her death, thirty years rolled over her head, only distinguished by increasing anguish. Some instances of her grief were romantically affecting. She frequently held in her livid hand her favourite watch from the morning to the night, to notice the spending even of every minute; so heavy to her was the lapse of time. If her husband by chance offered to take the watch, when he took his walk in the morning, she would shriek aloud for it; though her dwelling was contiguous to the clock in the parish *church*. She would peruse the letters of Lady Russel, twice each year, and contrast such misery with her own. In her work basket favourite epitaphs lay, which she daily read with bitter feeling; the one she preferred in Shakspeare, which ends with these cheering lines. Quiet consummation have, and renowned be thy grave. Every morning she was heard faintly and humbly to observe: "now I begin *another* day of misery;" and her last wish at every evening was, that she might be found a corpse in the morning. "On the anniversary of the month, and of the day in which her only daughter died, her sufferings ferings were still more poignant. She frequently passed both of them in her chamber. This custom gave a melancholy comfort to her troubled mind. It was a still and silent offering of respectful grief to the shade of her child." She not only absented herself from the genteel society in which she had moved, and relinquished the visits to the castle of Lord Mulgrave, to which she had been habituated, but she with a sigh, would invite the poorest and stupidest females from three adjacent hospitals to take tea at her table in mournful succession, one on each day. She was soothed by the sight of those unfortunate persons, whose wants she was able to relieve, and in whose simple tales of woe she could feel an interest without the necessity of those deep reflections, and without those tremors of false delicacy, to which a sympathetic condolence with the rich subjects the speaker. A daily present richly rewarded such humble companions. It was no common fall and no usual descent in life, to sink to the level of paupers, from an education amid the ladies of the nobility. The scenes of Mulgrave Castle, appeared to her like visions which had closed in a dark night.

Other instances of nervous maladies have occurred in the county of York. A student of Cambridge, (who has attained to a high rank in the church) when he had taken his first degree, sought in the country, the strength and the health which he had injured by his studies at the college. His person was large and muscular, his stomach digested rapidly, and his rosy complexion promised a superfluity of blood, of generous humours, and of rustic vigour. By the excessive intensity of his application, and by the natural irritability of his constitution, sleep forsook him for one or two nights; a depression of spirits, and a torpid melancholy arrested all his powers of mind and of body. His relations compelled him to ride on a horse in a retired village; but he was unable to guide the reins; a poor man was hired to lead it by the bridle. The scholar and the fine genius sat on the saddle with an idiotic gaze, motionless, torpid, insensible. Our minds shudder at such a picture. He recovered his health, however, in one season, and his love of science returned. But his habits of life continue uniform, tranquil, and retired. His robust figure is still liable to disturbed sleep, and the faintest sound awakes him.

The wife of a merchant had risen from humble life into unexpected opulence; her pride and her delicacy increased with her fortune. She had possessed in her youth no inconinconsiderable share of beauty, and the physician would have discovered in her figure every promise of long life and of perpetual health. The ambition of visiting her wealthier neighbours seized her, but she met with repulses and with disgrace. Discontended at her domestic and obscure situation, her soul brooded over it in indignant silence; a high nervous affection followed. Her husband prudently concealed her in her house, and compassionately administered to her food and medicine; yet the irritation of temper which the complaint had produced, caused her to dislike the presence of her husband. By the most gentle perseverance, she was restored to her family. A hectic flush in her complexion and a fiery keenness of eye are the only marks which attest to an intelligent observer the former existence of high nervous affection; as the transient view of a ruddy reflexion in the sky at midnight denotes to the natural philosopher the crater of an extinguished volcano.

A merchant, whose habit of body is thin and meagre, but who still possesses great activity and muscular energy, had engaged in expences, to which he deemed his fortune inadequate. A low nervous disorder harrassed him during twelve months; his powers of judgment were impaired; his fancy was heated; he talked incessantly, but in a low tone; the slightest circumstance fluttered and alarmed him. He was persuaded with difficulty to invigorate and brace his constitution by daily drinking a pint of wine, and by daily walks. A still life, seclusion in a village, and the minute and friendly attention which was paid to his comfort by his family, recovered him to all his former habits and all his youthful industry.

A magistrate, with whom the writer of this essay was acquainted, experienced from the dread of public censure on a delicate occasion, a bitter month of mental and nervous depression. His muscular and square frame trembled, his poetical imagination teemed with a thousand caprices, and gloomy apprehensions represented to him life as a certain scene of future disgrace. His prudent wife prevailed upon him to try the effect of a journey to his distant friends. The solitude of their own carriage, which moved slowly, the curiosity of the rural scenes in a remote country, which he had never visited, and the calm friendship of a surgeon who had been acquainted with all his family from his infancy, recruited his exhausted spirits, revived his attachments, and roused all his energies. No effects of the temporary disorder have remained.

The wife of a merchant, was, during a month, a victim of low nerves. She retired to her chamber, convinced that her dissolution was approaching! yet her frame was calculated for exercise and activity. Her diet had been moderate, and she indulged in walks daily and regular; success had crowned her with wealth, and her husband and children adored her. Her external fortune was splendid, but the state of her mind was thus suddenly dismal and desolate. A foolish physician had prescribed bleeding to her, though it would have proved inevitable death. A wiser practitioner requested her to eat nutritive soups, and rich viands of easy digestion. He gradually convinced her, that her disease was seated in the imagination, not in the stomach; and that her resolution to attempt her former habits of exercise would be crowned with success. She determined in one week upon the effort; she succeeded: she has never experienced a relapse, while the unhappy month appears a troubled dream.

A very studious character in the same neighbourhood, laboured for two months under a similar affection. It commenced in a high nervous complaint, and when it had reduced the sufferer to a low state of spirits, it fortunately disappeared. His constitution was radically strong, his feelings intense, his heart warm, and his attachments ardent. A predisposition to pensive melancholy attended him at school. When an old companion quitted the school to enter the stage of life in some distant city, he was heard to sigh deeply at the departure of a young friend, whom probably he should see no more; he repeatedly retired into the fields to weep the loss; and reclined on the bank of some lonely stream, keenly deplored the separation. Epitaphs and gloomy descriptions, and solemn or pathetic music, were the singular choice of his boyish years. A mother, too fond and too indulgent, awakened in his soul those affectionate raptures, to which the rude and harsh scenes of human life afford a terrible contrast. The mind was disqualified by such tenderness of treatment, to bear with manly firmness the opposition of rivals, the malice of enemies, the taunts of pride, and the ingratitude of friends. His father was capricious, alternately too severe and too lenient, too indignant, and too flattering. Such a conduct subjected the boy to the opposite passions of fear and of love, of humility and of pride. His master at the public school bestowed unbounded applause on his progress, and hailed his literary prospects: but he poured, by a wretched abuse of learned authority and of puritanical eloquence, into the inexperienced fancies of his pupils, pils, a dark and frightful vision of the future evils which they were instructed to expect. Hope, the sunshine of the youthful breast, was damped, and exchanged for dull despondency. Two tutors of fanatic opinions, dismal as those of the master, attempted in the college at which he was entered, to continue the despair and the gloom in which he had been educated. A series of disappointments, and a succession of sicknesses caused by excessive studies, blasted his fairest promise of eminence, and thus all the prizes which he had gained, and all the commendations of the president and the master, yielded no emolument and no promotion.

> The nightingale plunder'd, the mate-widow'd dove,
> The warbled complaint of the suffering grove;
> To my life as it varied, gave sentiment new,
> The object still changing, the sympathy true.

In a few years, when an accident gained him a living, his past misfortunes were augmented by the nervous disorder, which was aggravated by temporary causes of a domestic and private nature. He had refused to his body, by the protraction of his studies to a late hour, the necessary portion of sleep. His whole frame was shaken by a ride to too great a distance on a sultry Sunday, and by the sudden suppression of perspiration in a damp church. He ought to have retired to sleep immediately, but he was interrupted in his plan. Nature will be avenged on our inattention: he was afterwards unable to sleep during three tedious nights. His resolution was firm and his courage was unshaken, in this wakeful state of mental suffering; but his fancy was too active and too vivid to admit repose. His powers of recollection never failed him, though his judgment acted in a slow and languid manner. A nervous head-ache distressed him, and employed all his thoughts upon the manner in which it could be relieved. As he lived remote from the medical friend, in whose skill he placed his confidence, he prescribed a diet for his own case. The extremity of his pains would have reduced him to a skeleton, had he not supported nature with a nutritious diet. Opium would not only have agitated and injured his muscular frame, but would have been rejected by his stomach; while the breathing a vein would have weakened his constitution, and would not have alleviated his anguish. During the vigils of these three nights, his thoughts flowed with rapidity, and his reflections were desultory. But the sweetest sensation ever experienced, was the refreshment which he derived from the first hour of returning sleep on the fourth

 day.

day. It produced a profuse, but an invigorating perspiration. The whole body seemed awakened to new life, and to supreme delight. Every sense was soothed, and joy seemed diffused through all the limbs. No head-ache remained, no disagreeable symptom. Such a concussion, however, was given to his constitution, that his complaint was followed by depressed spirits. A prudent retirement to a healthful village, a temperate diet of fruits and jellies, and an abstinence from vinous and spirituous liquors, completed his restoration in one month. He has suffered from it no diminution in his bodily strength, and no loss in his mental powers. His profession conduces to his still and gentle life, and his religious principles forbid his indulgence in revenge or anger, or in any sensations but those of charity and benevolence.

A clergyman in our neighbourhood was disappointed in his dearest hopes by the death of a young lady, to whom he was engaged: and dejection succeeded. He ceased to love life, and invoked during many months, death as his expected friend. The solitude of a residence in a village increased the disorder, and his too active thoughts, which devoured his tranquillity, "made the meat they fed on." His companions gradually recalled him, by soothing his distress, to more active scenes. He travelled the tour of southern Europe. Fortune reserved for him a second matrimonial engagement, which rewarded him for his former sufferings. And all his advanced life has been adorned by numerous charities to the poor, by personal visits to their families in sickness, by donations of medicine or loans of money, and by the medical advice, which a medical education enabled him to give to the poor, or to his companions. "Haud ignarus mali, miseris succurrere discit:" while his recollection of his misery from the attack on his own nerves has softened a heart naturally kind, it has reanimated his attention to the afflicted. The attentive reader will learn, we trust, from this collection of nervous cases, the precautions and preventives which he should employ against the commencement of nervous disorders, and the diet, the arts, the measures which he should adopt, should they unhappily surprise him. Total change of scene and even manner of life is generally requisite, with absence from every object which strongly affects the patient; and the mind ought to be engaged by new attentions and new diversion. The medical treatment of such complaints is exceedingly well understood by really regular physicians of extended practice; such reside chiefly in

London.

London. So much delicacy, learned attention, and profound wisdom is there exhibited, in the management of such cases, that, I think, nothing can be added to their skill. The wisest caution in country towns is, to shun application to empirics of any description. Such a sufferer should be doubly cautious against nervous attacks, if his nature be very proud, or timid, or delicate. He should guard against sudden alarms in the night, or any sudden dangers in the day. He should inure his mind to bear scenes, the first view of which revolt his feelings and give a moral shock to his mind. He should habituate his fancy to contrasts. He should persevere in acquiring fortitude and firmness. He should seek no scenes of turbulence and indulge in no excess: but should his fortune permit, "keep the noiseless tenor of his way." Should he enjoy leisure, gentle study and successive exercise will be indispensible to his health. Should he act a part on the public stage of life, and enter on a crowded theatre of action, let him occasionally indulge in retirement, and frequently relax from his labours. The nervous constitution may suffer equally from an excessive tension of its fibres as from an excessive indolence. The fair sex in *our* age must be doubly warned against those habits which create nervous disorders. For though they are not as hereditary as the goût or the consumption, the child will acquire by an imitation of its nervous mother, a terrible predisposition to the complaint. Hence none of the wretched romances or novels, which convey to the irritable imaginations of girls ideas of false delicacy, of premature womanhood, of vanity and confidence, of pride and pomp, of parade and fashion, should ever pass into the hands of the young. Hence female education should wisely prepare them to encounter the difficulties and the cares of married or domestic life: and they should not be taught to expect an unvaried scene of ease and competence, or an incessant succession of "lovers, friends, and honourable characters." Such deceitful colourings and pictures of society, such "skies of gold and seas of amber," will inflame the female imagination, will inspire hopes of a happiness which they must never taste, and will conceal from them the miseries incident to human life. Evils thus unforeseen will shock their timorous nerves: deaths, bankruptcies, or sickness, will destroy their spirits, and sink them into despondency: hysteric and hectic affections will lead to convulsions, and palsies will introduce decripitude, dotage, and premature death.—We will not allow our imagination to to rove in pursuit of the other forms of nervous disease or debility: the present enumeration of them will be sufficiently terrific and appalling.

